# Responses of Secondary Inorganic Aerosols to Synergistic NO*_x_* and NH_3_ Emission Control Based on an Inversion Inventory

**DOI:** 10.3390/toxics14080657

**Published:** 2026-07-26

**Authors:** Xiaohui Du, Minghui Wei, Linglu Qu, Wei Tang, Chao Yu, Zhongzhi Zhang, Yang Yu, Yang Li

**Affiliations:** 1 Chinese Research Academy of Environment Sciences, Beijing 100012, China; duxh@craes.org.cn (X.D.); tangwei@craes.org.cn (W.T.); zhangzz@craes.org.cn (Z.Z.); yuyang@craes.org.cn (Y.Y.); liyang@craes.org.cn (Y.L.); 2 State Key Laboratory of Remote Sensing Science and Digital Earth, Aerospace Information Research Institute, Chinese Academy of Sciences, Beijing 100101, China; yuchao@aircas.ac.cn

**Keywords:** ammonia, NO*_x_*, secondary inorganic aerosol, joint inversion, emission inventory optimization, sensitivity analysis, Beijing−Tianjin−Hebei

## Abstract

Secondary inorganic aerosols (SIAs) are critical components of regional air pollution, yet uncertainties in bottom–up emission inventories lead to biases in the simulation of nitrate (PNO_3_^−^) and ammonium (PNH_4_^+^). This study utilizes a joint NO*_x_*−NH_3_ inversion inventory constrained by satellite observations to investigate the response characteristics of SIAs to precursor reductions in the Beijing–Tianjin–Hebei (BTH) region during July 2020. Results indicate that the a priori emission inventory significantly underestimated NH_3_ emissions in the BTH region, with a posteriori emission in cities such as Shijiazhuang and Handan increasing to 2–3 times the a priori levels, while NO*_x_* emissions were slightly underestimated. Sensitivity analysis conducted via Comprehensive Air Quality Model with extensions (CAMx)—Decoupled Direct Method (DDM) reveals that the sensitivities of PNO_3_^−^ and PNH_4_^+^ to precursor variations are higher in south–central BTH; specifically, the sensitivity of PNO_3_^−^ concentration to NO*_x_* emission abatement under the a posteriori inventory rises substantially compared with a priori estimates, with relative growth rates spanning 33% to 308% across the study domain. Scenario simulations demonstrate that synergistic NO*_x_* and NH_3_ control is the most effective strategy, reducing PNO_3_^−^ and PNH_4_^+^ concentrations by 2.27 ug/m^3^ and 0.77 ug/m^3^, respectively.

## 1. Introduction

In recent years, with the implementation of various air pollution prevention and control action plans, China has achieved remarkable success in reducing emissions of PM_2.5_ precursors such as SO_2_ and NO*_x_* [1]. Consequently, sulfate (PSO_4_^−^) concentrations in PM_2.5_ have gradually declined. Conversely, the fractional contributions of nitrate (PNO_3_^−^) and ammonium (PNH_4_^+^) to secondary inorganic aerosols (SIA) have exhibited a slight increase [2]. During severe winter haze episodes in the North China Plain, the contribution of SIAs to PM_2.5_ concentrations can even exceed 60% [3]. In such SIA-dominated pollution events, ammonia (NH_3_), functioning as a critical atmospheric alkaline precursor, plays a pivotal promoting role. Studies indicate that an elevation in atmospheric NH_3_ concentrations (0–200 ppb) can significantly accelerate the formation rate of secondary aerosols (by a factor of 2.0 to 2.5), with the enhancements in nitrate and ammonium being particularly pronounced [4].

Compared to other gaseous precursors that have seen substantial emission reductions, current mitigation and control measures for NH_3_ lag severely behind. Driven by intensive anthropogenic activities (particularly agricultural intensification), regional ammonia emissions in China have long persisted at a highly saturated level. Over the past three decades, annual ammonia emissions from Chinese croplands have been sustained at 3.3–4.0 Tg. These emissions are predominantly concentrated in Eastern, Central, and Northern China; notably, the average annual emissions in the Beijing–Tianjin–Hebei (BTH) region reach 1.2 Tg [5]. This persistently high NH_3_ emission provides abundant precursors for the heterogeneous reactions of SIA. Therefore, the rational control of NH_3_ emissions constitutes a core key to driving the steady decline of PM_2.5_ concentrations.

On the other hand, inherent uncertainties in current emission inventories constrain the quantitative evaluation of precursor mitigation effectiveness. Existing bottom–up NH_3_ emission inventories harbor substantial uncertainties. The uncertainty of NH_3_ emissions in Emissions Database for Global Atmospheric Research (EDGAR) inventory reaches 186–294% [6], while the uncertainty range for sectoral NH_3_ emissions in China within the Regional Emission inventory in ASia (REASv3) inventory is −79% to +311% [7]. Even in the BTH region, where emission statistics are relatively robust, regional inventory uncertainty remains between −32.6% and +38.7%, and summer NH_3_ emissions in certain cities have been found to be underestimated by ~5 times [5,8]. Such input errors severely compromise model performance in simulating secondary particulate matter. The formulation of scientifically sound NH_3_ mitigation strategies relies heavily on the precise assessment of emission reduction benefits by air quality models.

To mitigate emission inventory uncertainties, top–down inversion techniques integrating satellite remote sensing observations with chemical transport models have been extensively applied. Previous inversion studies primarily focused on constraining single pollutants [9,10]. However, strong chemical coupling effects exist between NO*_x_* and NH_3_ during the formation of ammonium nitrate. Prior research has demonstrated that adopting a joint inversion approach can effectively circumvent the compensatory biases introduced by single-species inversions [11]. Utilizing refined inventories with joint constraints to evaluate the non-linear response characteristics of secondary particles to precursor variations, and subsequently quantifying the impact of synergistic pollutant reductions on PM_2.5_ and its constituent concentrations, remains a critical component in developing precise co-control strategies.

In this study, employing a validated NO*_x_*−NH_3_ joint inversion inventory, we comprehensively investigate the impacts of emission baseline modifications on the simulation of SIAs and their associated sensitivities. Based on pre-optimization (a priori) and post-optimization (a posteriori) emission inventories, we focus on quantifying the dynamic evolution of regional emissions in the BTH region during July 2020. By comparatively analyzing the simulation discrepancies between the a priori and a posteriori inventories, this paper aims to elucidate how the correction of emission baselines alters the sensitivity characteristics of secondary particles (PNO_3_^−^ and PNH_4_^+^) to precursor emission reductions. Furthermore, we assess the effects of NO*_x_* and NH_3_ synergistic emission reductions on PM_2.5_ and secondary particle concentrations. This study aims to quantify the impacts of emission inventory biases on model simulation results and provide theoretical support for formulating regional air pollution control policies featuring synergistic pollutant reduction.

## 2. Data and Methods

### 2.1. Study Area and Simulation Configuration

**Figure 1:**
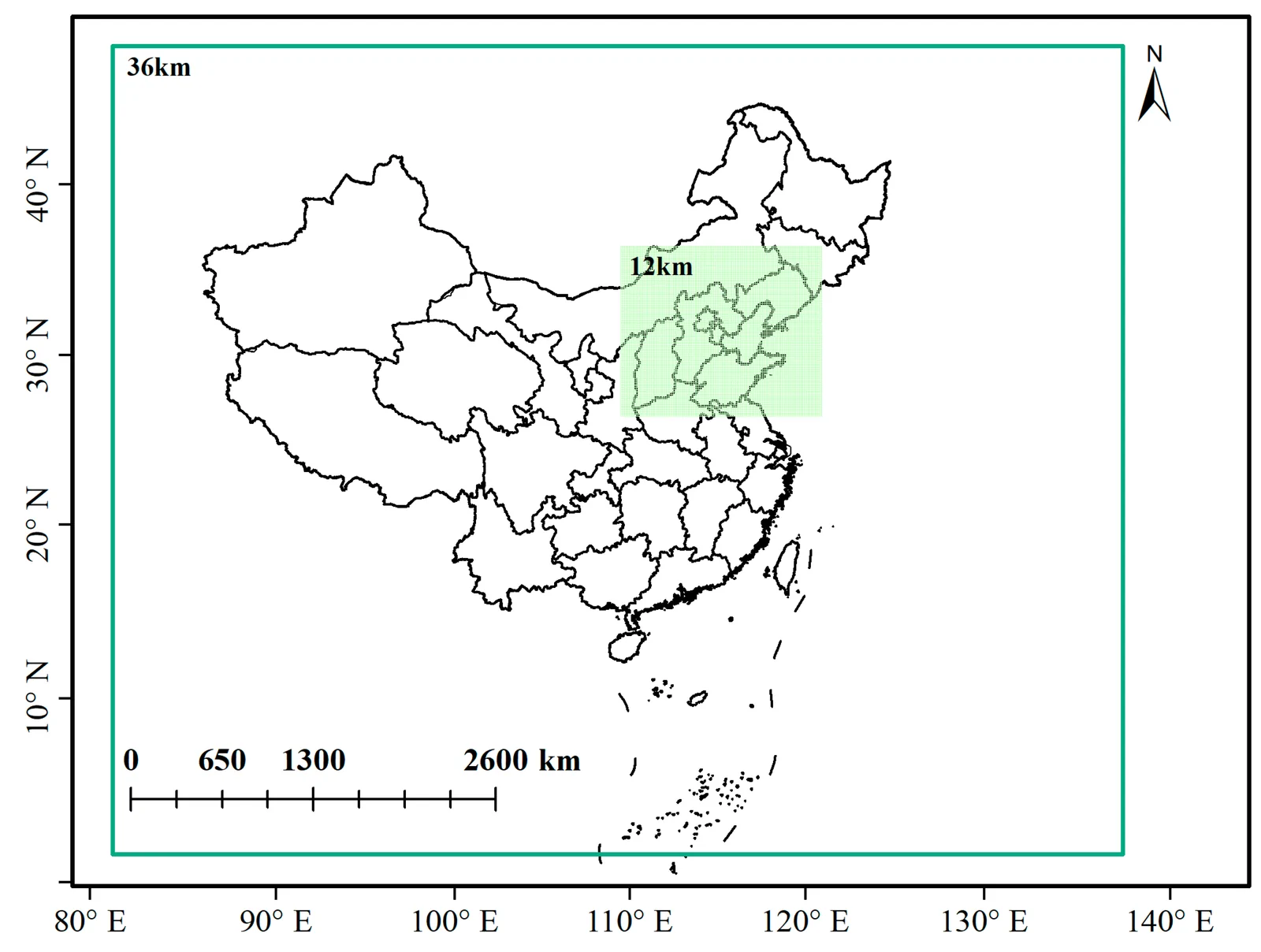
Simulation domain and nested grid settings with resolutions of 36 km and 12 km.

In this study, the Comprehensive Air Quality Model with extensions (CAMx v6.20; Ramboll, Novato, CA, USA) was applied to simulate the spatiotemporal variations of NO_2_, NH_3_, and SIAs [12]. As a three-dimensional Eulerian photochemical transport model, CAMx integrates atmospheric dynamics with complex chemical mechanisms, enabling the efficient handling of multi-scale grid nesting and coupled multi-pollutant simulations. This study adopted a double-nested grid system comprising an outer domain (D01) with a 36 km spatial resolution covering most of eastern China (105° E–130° E, 30° N–46° N) and an inner nested domain (D02) with a 12 km resolution focused on the BTH region and its surrounding areas (Figure 1). The model is divided into 20 vertical layers extending from the surface to the tropopause. The SAPRC99 gas-phase chemical mechanism was employed [13]. To quantify the non-linear response of precursor concentrations to emission perturbations, the Decoupled Direct Method (DDM) built into CAMx was utilized to calculate the sensitivity coefficients of concentrations to emissions, thereby analyzing the emission-concentration response relationships in the BTH region [14]. Given that ammonia volatilization is highly pronounced in summer, the simulation was conducted for a typical summer period in July 2020.

### 2.2. Emission Inventories

#### 2.2.1. A Priori Emission Inventory

As a key input parameter, the a priori pollutant emission inventory is an important factor governing the accuracy of top–down constraints. In this study, the spatial distribution of the a priori inventory configured for the master grid (36 km × 36 km) and the nested grid (12 km × 12 km) was based on the Multi-resolution Emission Inventory for China (MEIC 2020) [15]. The total emissions of pollutants, including NO*_x_*, SO_2_, VOCs, and NH_3_, were subsequently revised based on localized emission data for the BTH region. The original MEIC inventory has a spatial resolution of 0.25° × 0.25° on a regular latitude-longitude grid under a geographic coordinate system. This is inconsistent with the equidistant grid under the projected coordinate system configured in the CAMx. To satisfy model input requirements and eliminate discrepancies in both projection systems and grid resolutions, the emission data on the geographic latitude-longitude grid were spatially reallocated. This reallocation was performed using a custom-developed script based on a sector-specific spatial proxy approach. For instance, population density, industrial land use, and cropland distribution were utilized to allocate residential, industrial, and agricultural emissions, respectively. This method ensured total mass conservation while converting the data into the format required for regional grids under the projected coordinate system, matching the simulation resolutions of 36 km and 12 km in CAMx.

#### 2.2.2. Posterior Emission Inventory via NO*_x_*−NH_3_ Synergistic Constraint Inversion

In this study, the a priori emission inventory was constrained and optimized based on a jointly constrained NO*_x_*−NH_3_ inversion algorithm. This joint inversion method utilizes Infrared Atmospheric Sounding Interferometer (IASI) satellite-observed NH_3_ column densities and TROPOspheric Monitoring Instrument (TROPOMI)-observed NO_2_ column densities as dual constraints. Utilizing the discrete Kalman filter (DKF) method combined with the spatial response matrix between pollutant concentrations and emissions simulated by CAMx−DDM, the algorithm resolves the non-linear relationship between atmospheric pollutants and emission through multiple iterations, thereby quantifying potential biases in the emission inventory and retrieving an updated emission. Compared to conventional independent inversions, this method significantly alleviates systematic biases in the a posteriori emission by accounting for the mutual feedback between NH_3_ and NO*_x_* emissions and NO_2_ and NH_3_ concentrations. The joint inversion framework (Appendix A) and its performance have been fully validated and systematically evaluated in our previous study [11].

**Figure 2:**
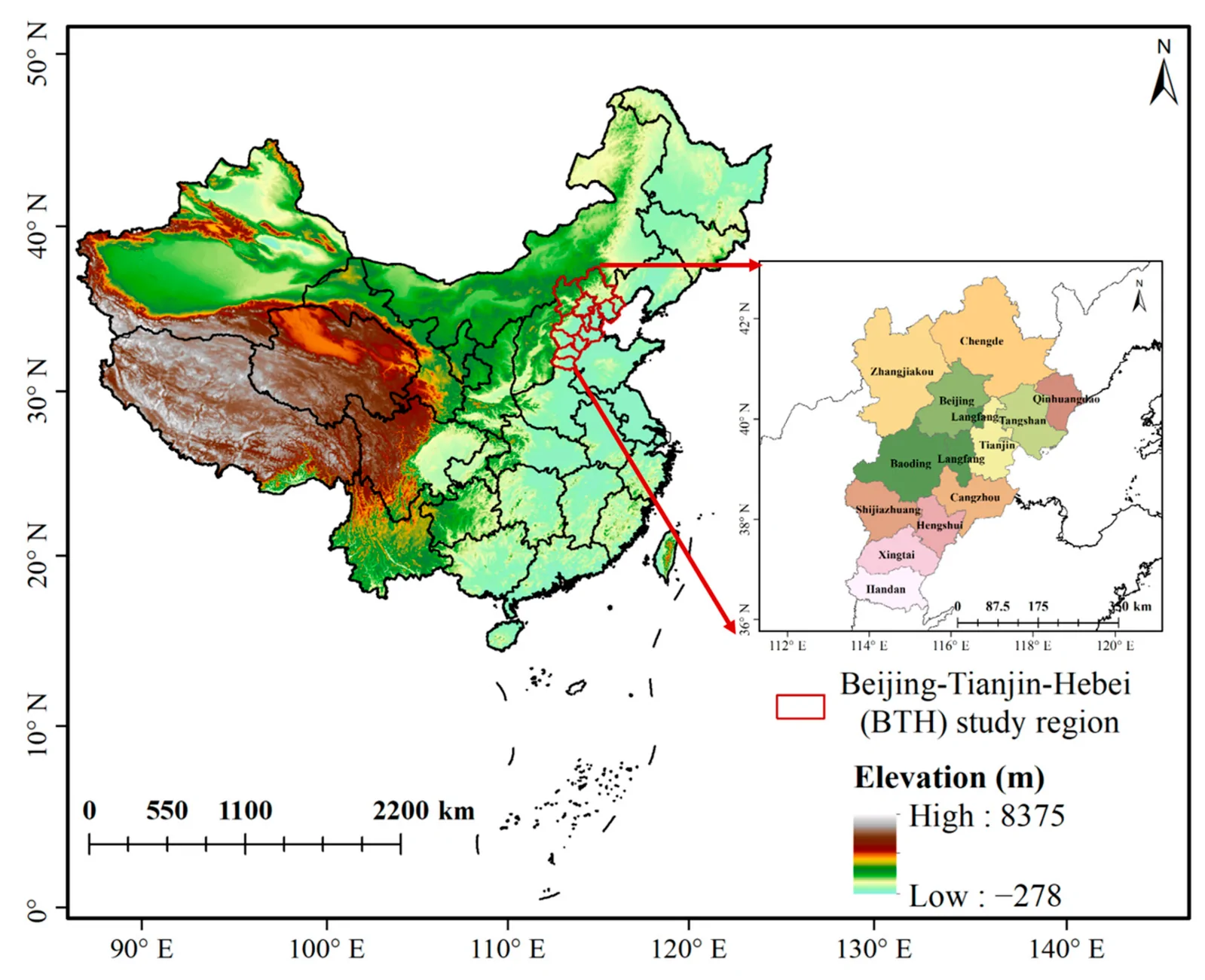
Regional division of the Beijing–Tianjin–Hebei region for CAMx−DDM simulation.

Following the previously established joint inversion methodology, this study constrained the a priori emission inventory for July 2020. During the inversion process, the BTH region was divided into 12 distinct sub-regions for separate emission constraints, including Beijing, Tianjin, Baoding, Langfang, Cangzhou, Shijiazhuang, Hengshui, Xingtai, Handan, Tangshan, Qinhuangdao, Zhangjiakou, and Chengde, with Baoding and Langfang merged into a single region (Figure 2).

### 2.3. Scenario Simulation Design

To quantitatively evaluate the non-linear response characteristics of SIAs to precursor emission reductions, and to explore the impact of emission inventory optimization on mitigation assessments, this study designed various emission perturbation scenarios for July 2020 (Table 1). Across all scenario simulations, the meteorological fields, initial conditions, and boundary conditions were kept identical. Baseline scenarios were first established based on the a priori and a posteriori emission inventory, respectively. On this basis, to compare the response characteristics of ${\mathrm{PNO}}_{3}^{-}$ and ${\mathrm{PNH}}_{4}^{+}$ concentrations to single precursor reductions, and considering that NH_3_ emissions in the BTH region have remained persistently high, wide-ranging reduction ratios (30–70%) were applied exclusively to NH_3_ emissions in the S1 scenario to investigate the impacts of NH_3_ on secondary particles under extreme control conditions. Meanwhile, in accordance with the Action Plan for Carbon Dioxide Peaking Before 2030, under which NO*_x_* emissions are projected to decrease by 20–30% by 2030, an independent NO*_x_* reduction scenario (S2) was established. Finally, to analyze the environmental impacts of multi-pollutant synergistic control, a synergistic emission reduction scenario (S3) was set up, which simultaneously reduced NO*_x_* and NH_3_ emissions by 30% and 70%, respectively. All the aforementioned scenarios were simulated under both the a priori and a posteriori emission inventory. By comparing the discrepancies in concentration responses under identical perturbations across different inventories, we quantified the potential systematic biases in emission reduction assessments caused by emission inventory uncertainties.

## 3. Results

### 3.1. Spatial and Temporal Differences in Emissions Before and After Constraint Optimization

**Figure 3:**
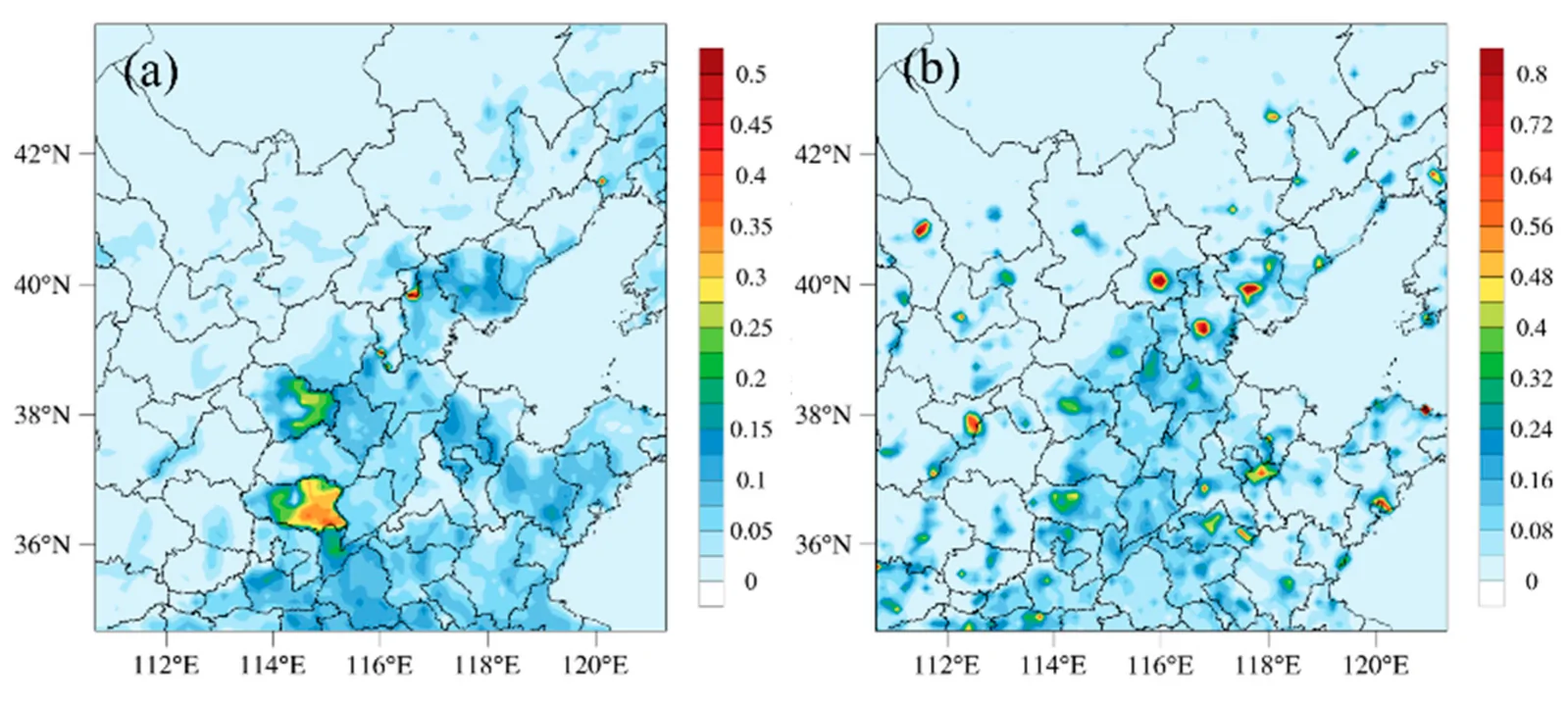
Spatial distribution of a priori precursor emissions for the BTH region in July 2020. (**a**) Spatial distribution of NH_3_ emissions; (**b**) Spatial distribution of No*x* emissions.

In this section, a localized emission inventory was adopted as the a priori input, based on which subsequent inversion and optimization were conducted. The prior and posterior emission inventories were evaluated by comparing the simulated NH_3_ and NO_2_ column concentrations with satellite observations, as shown in Figure S1 in the Supplementary Material. Figure 3 illustrates the spatial distribution of the a priori emission inventory over the BTH region, where the total monthly NH_3_ emissions amounted to 75.54 kilotons (kt) and the total monthly NO*_x_* emissions reached 100.06 kt. NH_3_ emissions are significantly influenced by intensive agricultural activities, with high-emission areas being highly concentrated. In contrast, NO*_x_* emissions are dominated by industrial and transportation sources, exhibiting a typical distribution of discrete point-like hotspots.

Previous studies have demonstrated that both NH_3_ emissions and column densities remain at relatively high levels throughout the year during summer [16]. To ensure the representative nature of the research findings while considering the computational burden of the model during joint inversions, July was selected as the typical period of summer. The joint inversion method was then applied to conduct a monthly joint inversion of the a priori NH_3_ and NO*_x_* emissions over the BTH region.

According to the change rates of NH_3_ and NO*_x_* emissions in each sub-region before and after the inversion (Table 2), NH_3_ emissions in most cities exhibited a significant increase following the joint inversion. In July 2020, the total a posteriori NH_3_ emissions in the BTH region were 273.20 kt. In most areas, the a posteriori NH_3_ emissions increased by more than 100% compared to the a priori baseline, with Shijiazhuang and Handan experiencing increases exceeding 200% in July. This indicates that the a priori emission inventory significantly underestimated the summer NH_3_ emissions in the BTH region.

**Figure 4:**
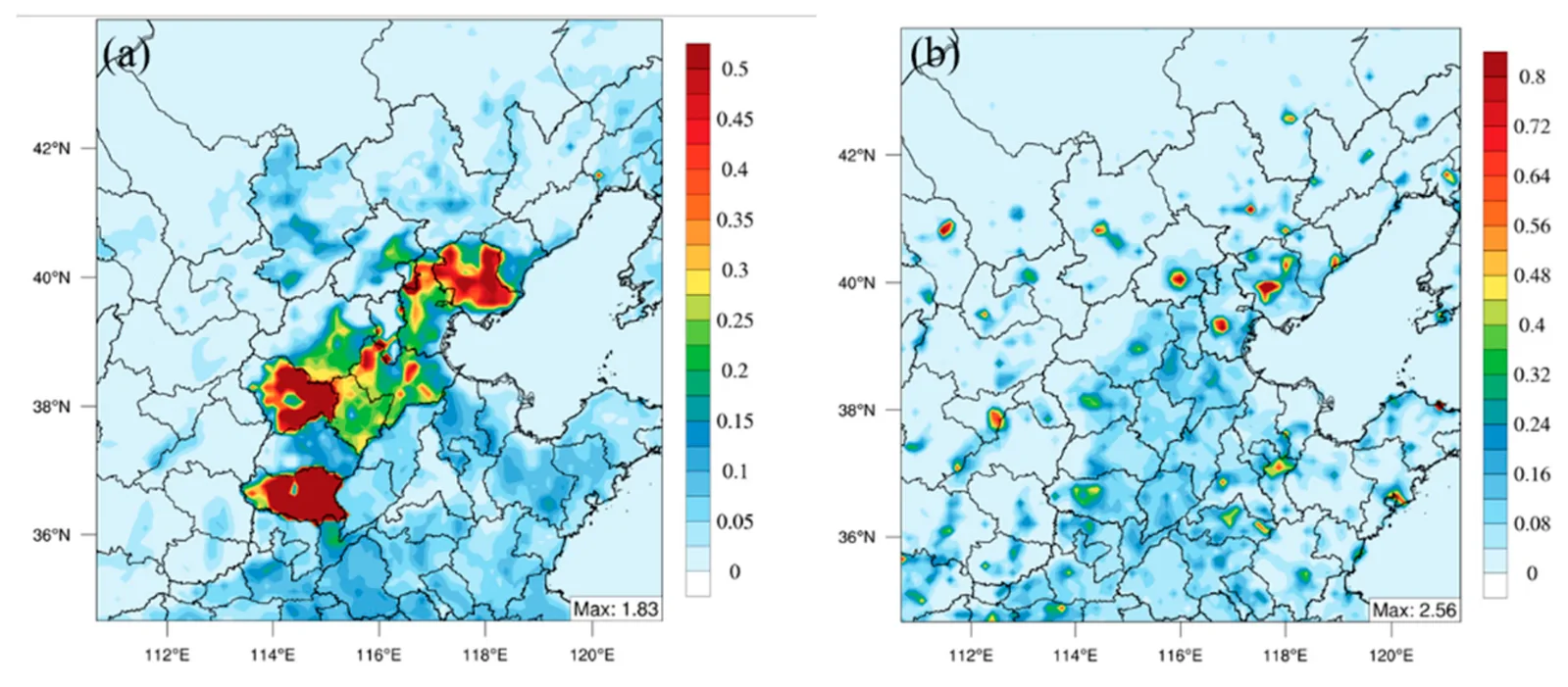
Spatial distribution of a posteriori precursor emissions in July 2020. (**a**) Spatial distribution of NH_3_ emissions; (**b**) Spatial distribution of NO*x* emissions.

Compared with the variations in NH_3_ emissions, NO*_x_* emissions exhibited relatively small changes before and after joint inversion. While changes in most cities were modest, Tangshan and Qinhuangdao showed substantial increases of 35.59% and 35.06%, respectively, and Chengde and Zhangjiakou exhibited even larger relative increases (82.91% and 86.65%), albeit from much lower prior emission levels. The total posterior NO*_x_* emission of the BTH region reached 109.65 kt, with an overall increase of approximately 9.60% compared with the prior inventory. The relative changes in city-level posterior BTH emissions ranged from −15.43% to 86.65% of the a priori precursor emission inventory (Figure 4).

### 3.2. Variations in Component Sensitivity Under Different Emission Inventories

The impacts of NH_3_ and NO*_x_* emission changes on PNO_3_^−^ and PNH_4_^+^ concentrations are not simply linear. To quantify the non-linear response of PNO_3_^−^ and PNH_4_^+^ concentrations to variations in gaseous precursor emissions, this section, following the optimization of NH_3_ and NO*_x_* emission inversions, analyzes the sensitivity changes of PNO_3_^−^ and PNH_4_^+^ concentrations to NH_3_ and NO*_x_* emissions before and after the emission inversions based on CAMx−DDM simulations.

The sensitivity coefficient $S_{i,j}$ simulated by CAMx−DDM represents the response of product concentrations induced by changes in precursor emissions, which is mathematically defined as:

(1) $$S_{i,j}=E_{j}\frac{\partial C_{i}}{\partial E_{j}}=\frac{\partial C_{i}}{\partial \varepsilon_{j}}$$

where $C_{i}$ is the concentration of secondary pollutant i (PNO_3_^−^ or PNH_4_^+^), and $E_{j}$ is the emission of precursor pollutant j (NH_3_ or NO*_x_*), and $\partial \varepsilon_{j}$ denotes the emission reduction fraction (ranging from 0 to 1). The coefficient $S_{i,j}$ is a semi-normalized sensitivity as directly output by the CAMx−DDM module, with the same physical units as $C_{i}$ (i.e., μg/m^3^ for particulate species). Physically, $S_{i,j}$ represents the change in $C_{i}$ corresponding to a 100% change (i.e., from $\varepsilon_{j}=0$ to $\varepsilon_{j}=1$) in precursor emissions. A positive $S_{i,j}$ indicates that an increase in emissions leads to an increase in concentrations, whereas a negative value represents an inhibitory response. The four sensitivity indicators analyzed in this section—$S_{{NH}_{3}}^{{{PNO}_{3}}^{-}}$, $S_{{NH}_{3}}^{{PNH}_{4}^{+}}$, $S_{{NO}_{x}}^{{PNO}_{3}^{-}},\;S_{{NO}_{x}}^{{PNH}_{4}^{+}}$—represent the sensitivities of PNO_3_^−^ and PNH_4_^+^ to NH_3_ and NO*_x_* emissions, respectively.

#### 3.2.1. PNH_4_^+^ and PNO_3_^−^ Sensitivity to NH_3_ Emission Change

Based on the CAMx−DDM simulation results under the a posteriori emission inventory, the sensitivities of PNO_3_^−^ and PNH_4_^+^ concentrations to NH_3_ emission variations in the BTH region exhibit pronounced spatial heterogeneity. Table 3 displays the mean sensitivities of PNO_3_^−^ and PNH_4_^+^ concentrations to NH_3_ emission changes in each city under the a posteriori emission condition, which characterizes the spatial distribution of these sensitivities across the region. These city-level results represent the degree of influence of precursor emission changes in each sub-region on the variation of PNO_3_^−^ and PNH_4_^+^ concentrations.

Regarding the impacts of NH_3_ emissions on secondary particles, the sensitivities of PNO_3_^−^ and PNH_4_^+^ concentrations to NH_3_ emissions exhibit a spatial pattern of being high in plains and low in mountainous areas. The high-value areas are mainly distributed in the plain cities of south–central Hebei Province, particularly in Handan, Shijiazhuang, and Cangzhou, indicating that secondary particle formation processes in these regions are more sensitive to NH_3_ emission perturbations. Among the studied cities, Handan exhibits the highest sensitivity of PNO_3_^−^ concentration to NH_3_ emissions, with an a posteriori sensitivity coefficient of 1.60 ug/m^3^ and an a priori value of 1.18 ug/m^3^ in summer. By contrast, the northwestern mountainous areas (such as Zhangjiakou and Chengde) show lower sensitivities, where the responses of PNO_3_^−^ and PNH_4_^+^ concentrations to NH_3_ emission variations are relatively weak.

Based on the a posteriori estimates, the overall sensitivities of PNH_4_^+^ concentration to NH_3_ emissions is smaller than that of PNO_3_^−^, with the urban mean ratios ranging from approximately 30.88% to 63.50% across the cities. In summer, the urban mean sensitivities range from 0.14 ug/m^3^ to 0.50 ug/m^3^, and the high-value areas primarily appear in Handan, Shijiazhuang, and Qinhuangdao. The spatial distribution of PNH_4_^+^ sensitivity to NH_3_ emissions does not completely overlap with the spatial pattern of NH_3_ emissions. This indicates that, besides local emissions, the response of PNH_4_^+^ concentration to NH_3_ emission variations may be simultaneously constrained by regional acidic precursor emissions, gas-particle partitioning conditions, and meteorological factors [17].

#### 3.2.2. PNH_4_^+^ and PNO_3_^−^ Sensitivity to NO*_x_* Emission Change

Regarding the impacts of NO*_x_* emissions on secondary particles, the high-value areas of the sensitivities of PNO_3_^−^ and PNH_4_^+^ concentrations to NO*_x_* emissions are mainly concentrated in the south–central regions of Hebei Province, such as Shijiazhuang, Handan, Baoding, and Langfang. Among them, the sensitivity of PNO_3_^−^ concentration to NO*_x_* emissions in Shijiazhuang reaches 1.39 ug/m^3^ in summer; in contrast, the northwestern mountainous areas such as Zhangjiakou and Chengde exhibit overall lower sensitivities, where the responses of PNO_3_^−^ and PNH_4_^+^ concentrations to NO*_x_* emission perturbations are relatively limited (Table 4). For the sensitivity of PNH_4_^+^ concentration to NO*_x_* emissions, its spatial distribution is generally similar to that of PNO_3_^−^, but the overall values and extent of influence are smaller (with sensitivity values below 0.5 ug/m^3^), indicating a relatively weaker response intensity of PNH_4_^+^ concentration to NO*_x_* emission variations.

#### 3.2.3. Differences in PNH_4_^+^ and PNO_3_^−^ Sensitivities Between a Priori and a Posteriori Emission Inventory

**Figure 5:**
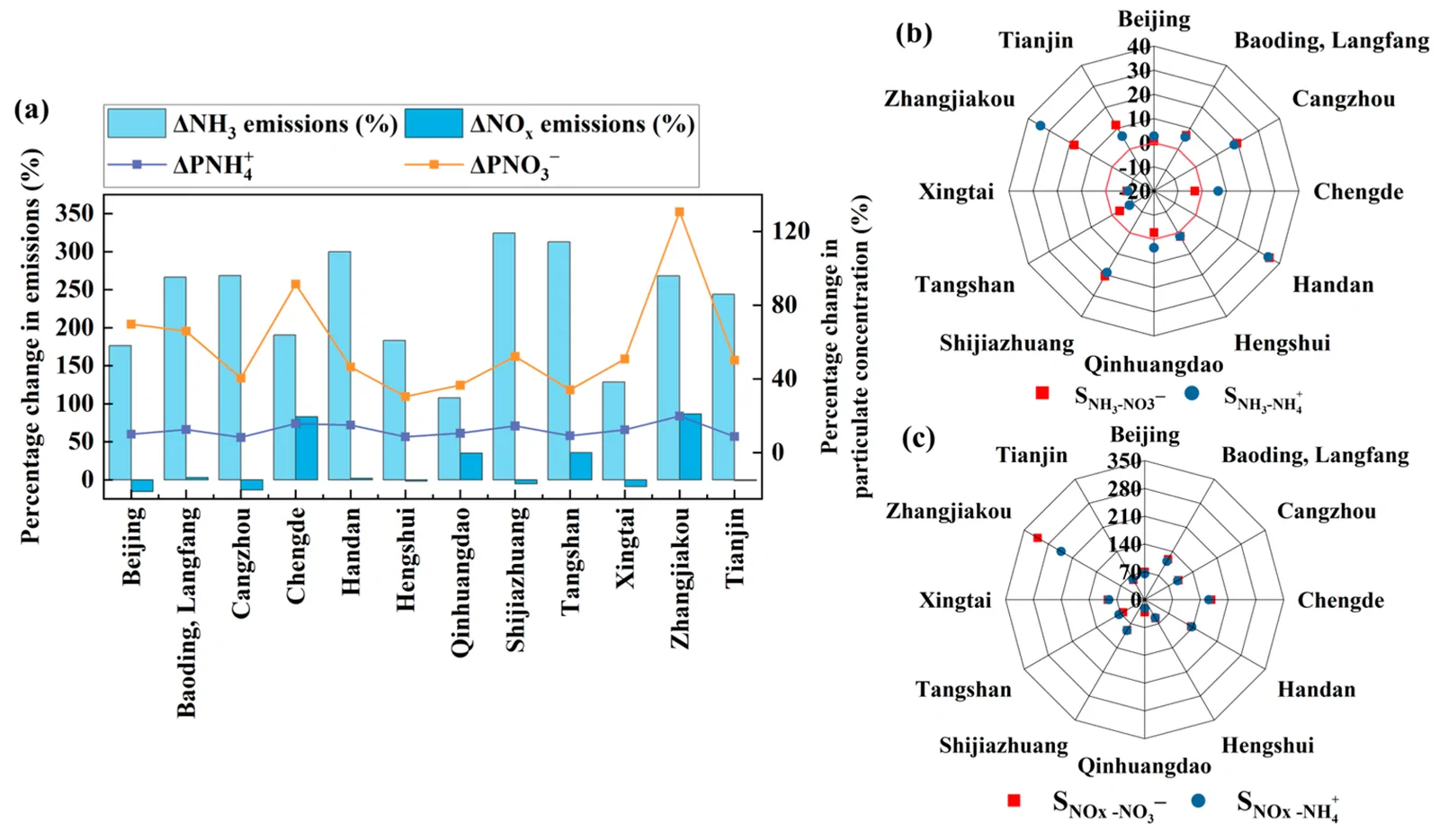
Rates of change in emissions, concentrations, and sensitivity coefficients between a priori and a posteriori simulation for each city in the BTH region in July 2020. (**a**) Relative change rates from prior to posterior simulations for NH_3_ and NO*x* emissions, and PNO_3_^−^ and PNH_4_^+^ concentrations. (**b**) Sensitivity change rate of PNO_3_^−^ and PNH_4_^+^ concentrations to NH_3_ emissions (red dots for the sensitivity coefficients of PNO_3_^−^ to NH_3_ emissions; blue dots for the sensitivity coefficients of PNH_4_^+^ concentrations to NH_3_ emissions). (**c**) Sensitivity change rate of PNO_3_^−^ and PNH_4_^+^ concentrations to NO*x* emissions (red dots for the sensitivity coefficients of PNO_3_^−^ to NO*x* emissions; blue dots for the sensitivity coefficients of PNH_4_^+^ concentrations to NO*x* emissions).

To evaluate the impact of emission inversion on the response relationships between PNO_3_^−^ and PNH_4_^+^ concentrations and precursor emissions, Figure 5 compares the changes in the sensitivity of PNO_3_^−^ and PNH_4_^+^ concentrations to NH_3_ and NO*_x_* emissions across different cities under the a priori and a posteriori emission condition. In the BTH region, the NH_3_ emissions have significantly increased in all cities, with a growth rate ranging from 107.61% to 324.46% (Figure 5a). However, the NO*_x_* emissions showed different trends. Cities such as Beijing, Shijiazhuang, and Hengshui experienced a decrease, while Zhangjiakou and Chengde showed a significant increase. Compared with the simulation results based on the a priori emission, the concentrations of PNO_3_^−^ and PNH_4_^+^ increased in all cities. Among them, the increase in PNO_3_^−^ concentration was the most significant, approximately 130.74% (in Zhangjiakou).

In summer, the sensitivities of PNO_3_^−^ and PNH_4_^+^ concentrations to a posteriori NH_3_ emissions in most cities of the BTH region are higher than their a priori simulated values, with a maximum increase of 36% (Figure 5b). The increases in PNO_3_^−^ sensitivity to NH_3_ emissions in Handan, Shijiazhuang, and Zhangjiakou all exceed 20%. Notably, the sensitivities of PNO_3_^−^ and PNH_4_^+^ concentrations to a posteriori NH_3_ emissions in some cities are lower than those in the a priori simulation, particularly in Xingtai and Shijiazhuang, where the sensitivities of both PNO_3_^−^ and PNH_4_^+^ to a posteriori NH_3_ emissions are smaller than their a priori counterparts.

The sensitivities of PNO_3_^−^ and PNH_4_^+^ concentrations to NO*_x_* emission under the a posteriori inventory are all higher than the a priori simulated values (Figure 5c). The increase in the sensitivity of PNO_3_^−^ concentration to NO*_x_* emission varied between 33% and 308% across different cities, with the largest increase occurring in Zhangjiakou. The sensitivity of PNH_4_^+^ concentration to NO*_x_* emission also increased in all cities, with changes ranging from 18% to 250%; the highest increase was observed in Zhangjiakou, while Qinhuangdao showed the smallest increase. The sensitivity of PNO_3_^−^ concentration to NO*_x_* emission in Zhangjiakou increased the most significantly, with an increase of over 300% (Table 4), which can be attributed to the relatively low baseline PNO_3_^−^ concentration in Zhangjiakou that amplifies the relative reduction rate under the same absolute perturbation. This indicates that the a priori inventory generally underestimates the mitigation efficacy of NO*_x_* emission reductions on secondary particulate matter during summer. At the same time, the negative sensitivities observed in some cities under a priori conditions turn positive under a posteriori conditions. Such deviations in the emission inventory not only affect the evaluation of the response intensity of secondary particle formation to precursor emission changes, but, more importantly, determine the trend of increase or decrease in concentrations.

### 3.3. Control Effect of Combined NO_x_ and NH_3_ Synergistic Emission Reduction

**Figure 6:**
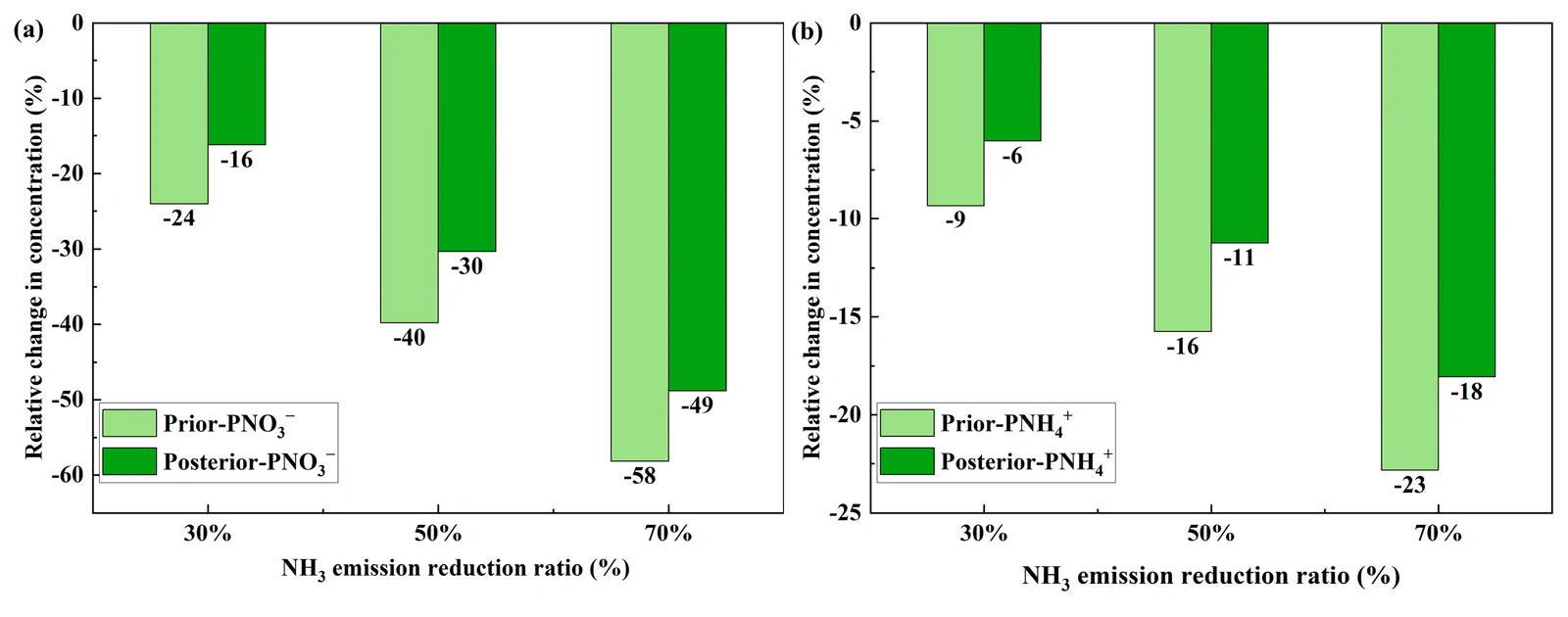
Response of PNO_3_^−^ and PNH_4_^+^ concentrations to varying NH_3_ emission reduction ratios under Scenario 1 in the BTH region. (**a**) Reduction rates of PNO_3_^−^ concentrations (in %) under 30%, 50%, and 70% NH_3_ emission reduction scenarios. (**b**) Reduction rates of PNH_4_^+^ concentrations (in %) under 30%, 50%, and 70% NH_3_ emission reduction scenarios.

Figure 6 presents the simulation results of the S1 scenario in July 2020. Based on the post-inversion simulation results, both surface concentrations of PNO_3_^−^ and PNH_4_^+^ exhibit decreasing trends as NH_3_ emissions are reduced. Simulations are carried out under three NH_3_ reduction ratios of 30%, 50%, and 70%. The decline magnitudes of surface PNO_3_^−^ and PNH_4_^+^ concentrations increase alongside higher NH_3_ reduction ratios, and the growth rate of concentration reduction slightly rises when the NH_3_ emission reduction ratio exceeds 50%, indicating non-linear response characteristics. Corresponding to the NH_3_ reduction ratios of 30%, 50%, and 70%, the decreases in surface PNO_3_^−^ concentrations in July are 16.14%, 30.14%, and 48.91%, respectively, while those in surface PNH_4_^+^ concentrations are 5.97%, 11.18%, and 18.10%, respectively.

Under identical reduction levels, the decrease in surface PNO_3_^−^ concentration consistently exceeds that of PNH_4_^+^, which is in agreement with previously reported results [18]. At the same reduction ratio, when the a priori emissions are used as the baseline for emission reductions, the absolute reductions in surface PNO_3_^−^ and PNH_4_^+^ concentrations are generally larger than those in the scenarios based on the a posteriori emission. The a posteriori emission scenario has a higher initial NH_3_ emission level, providing a stronger buffering capacity to the environment against NH_3_ reductions; consequently, the decreases in PNO_3_^−^ and PNH_4_^+^ are relatively smaller across all emission reduction stages.

The mitigation effects of independent NH_3_ and NO*_x_* reductions were compared with those of their synergistic reductions (where the 70% NH_3_ reduction scenario in S1 was selected for comparison with S2 and S3). Simulation results based on the a posteriori emission inventory show that, under the synergistic NH_3_ and NO*_x_* reduction scenario, the reductions in PNO_3_^−^ and PNH_4_^+^ concentrations (2.27 ug/m^3^ for PNO_3_^−^, and 0.77 ug/m^3^ for PNH_4_^+^) are greater than those under the independent NO*_x_* reduction scenario (0.87 ug/m^3^ for PNO_3_^−^, and 0.25 ug/m^3^ for PNH_4_^+^). In the simulation using the prior inventory data, the reduction efficiency of PNH_4_^+^ was relatively weak because the prior emissions underestimated the actual ammonia emissions. The lower baseline ammonia concentration limited the potential for PNH_4_^+^ formation, resulting in limited space for the decrease of PNH_4_^+^ under emission control. Under the current background of continuous NO*_x_* emission reductions, strengthening NH_3_ control can further promote the decline of surface PNO_3_^−^ and PNH_4_^+^ concentrations (Table 5).

## 4. Discussion

The underestimation of NH_3_ emissions in the a priori inventory for the BTH region, particularly during summer, is consistent with previous studies indicating that high temperatures in North China facilitate intensive volatilization, resulting in NH_3_ levels that typically exceed traditional estimates [8,19]. Following inventory optimization, the sensitivity of PNO_3_^−^ concentrations to NO*_x_* emissions exhibited obvious growth across all cities, with relative growth rates ranging from 33% to 308%, which indicates that the BTH atmosphere is in a more pronounced ‘NH_3_-rich’ state than previously estimated [20,21]. In such an environment, the formation of nitrate is limited by the availability of nitric acid; consequently, the a posteriori NO*_x_* emission controls are more effective than predicted by the a priori inventory. Conversely, the non-linear response of SIAs to NH_3_ reductions—particularly when reductions exceed 50%—suggests that the efficiency of NH_3_ control will increase as the atmosphere shifts toward NH_3_-limited conditions. Results from the synergistic reduction scenario (S3) further emphasize that while NO*_x_* reduction remains the primary focus of current management, simultaneous NH_3_ abatement can significantly amplify the decline of secondary particles. Future policies should account for these sensitivity characteristics to establish city-specific and pollutant-specific mitigation targets.

It should be noted that the current sensitivity estimates encompass inherent uncertainties due to the thermodynamic coupling between PSO_4_^2−^ and the NH*_x_*–NO_3_ system [17,22]. In this joint inversion, we primarily focused on NO*_x_*–NH_3_ interactions under a fixed SO_2_ baseline, given that anthropogenic SO_2_ emissions and ambient PSO_4_^2−^ concentrations across the BTH region have plummeted since the implementation of China’s Clean Air Actions in 2013 [1,23]. The drastic reduction in sulfate has substantially weakened its competitive consumption of NH_3_, shifting the region into a pronounced ‘NH_3_-rich’ state. Under such conditions, the formation pathways of regional nitrate and ammonium are predominantly governed by NO*_x_* and NH_3_ interactions [24]. Nevertheless, treating SO_2_ as a static background inevitably introduces some ambiguity into the quantitative sensitivity coefficients. Future studies should consider developing a multi-species joint inversion framework that incorporates dynamic SO_2_ adjustments to further constrain modeling uncertainties.

## 5. Conclusions

To mitigate the inherent uncertainties in bottom–up emission inventories, this study optimized the emission for the BTH region using a joint NO*_x_*−NH_3_ inversion constrained by satellite observations. By integrating CAMx−DDM sensitivity analysis with multi-scenario simulations, we systematically evaluated how the correction of emission constraints alters the formation of SIAs and the efficacy of precursor control strategies. The main conclusions are summarized as follows:

The joint inversion results indicate that bottom–up inventories significantly underestimate summer NH_3_ emissions in the BTH region. After satellite-based optimization, the total a posteriori NH_3_ emission reached 273.20 kt, compared to 75.54 kt in the a priori inventory, with key cities such as Shijiazhuang and Handan experiencing increases exceeding 200%. In contrast, NO*_x_* emissions exhibited relatively minor changes, with the total posterior value reaching 109.65 kt.

The sensitivities of PNO_3_^−^ and PNH_4_^+^ to precursor emissions exhibit a distinct spatial distribution pattern, characterized by higher values in the plains and lower values in the northwestern mountainous areas. South–central plain cities, particularly Handan and Shijiazhuang, show the most pronounced response to variations in NH_3_ and NO*_x_* emissions.

Simulations of emission reduction scenarios using the a posteriori emission inventory show that the effectiveness of NO*_x_* reduction in lowering PNO_3_^−^ concentrations rises by 33% to 308% compared with results derived from the a priori emission inventory. This suggests that, due to the previous underestimation of ammonia levels, traditional models may have significantly underestimated the environmental benefits of nitrogen oxide mitigation.

Scenario simulations demonstrate that, while SIAs exhibit a non-linear response to precursors, the synergistic reduction of NH_3_ and NO*_x_* achieves the most significant results, decreasing PNO_3_^−^ and PNH_4_^+^ concentrations by 2.27 μg/m^3^ and 0.77 μg/m^3^, respectively.

## Figures and Tables

**Table 1:** Emission reduction scenarios.

Scenario	Emission Reduction Configuration	
Base	A priori baseline emissions	A posteriori baseline emission
S1	Independent NH_3_ reduction of 30%, 50%, and 70%	Independent NH_3_ reduction of 30%, 50%, and 70%
S2	Independent NO*x* reduction of 30%	Independent NO*x* reduction of 30%
S3	Combined reduction of 70% NH_3_ and 30% NO*x*	Combined reduction of 70% NH_3_ and 30% NO*x*

**Table 2:** Relative changes in posterior emissions compared with prior emissions (Emissions units: kilotons; relative change = (Posterior − Prior)/Prior × 100%.).

City	NH_3_	NO*_x_*
	Prior/Posterior (Relative Change, %)	Prior/Posterior (Relative Change, %)
Beijing	3.67/10.13 (176.32)	9.41/7.96 (−15.43)
Baoding, Langfang	5.70/20.87 (266.35)	12.01/12.37 (3.06)
Cangzhou	6.59/24.26 (268.42)	13.73/11.87 (−13.53)
Chengde	2.86/8.31 (190.36)	5.10/9.33 (82.91)
Handan	17.65/70.59 (299.90)	10.25/10.45 (1.90)
Hengshui	5.18/14.67 (183.35)	5.16/5.06 (−1.77)
Qinhuangdao	2.19/4.56 (107.61)	3.44/4.65 (35.06)
Shijiazhuang	10.81/45.90 (324.46)	8.26/7.82 (−5.34)
Tangshan	7.96/32.86 (312.85)	11.13/15.10 (35.59)
Xingtai	3.83/8.75 (128.59)	7.62/6.93 (−9.16)
Zhangjiakou	3.99/14.70 (268.30)	4.94/9.23 (86.65)
Tianjin	5.12/17.62 (243.91)	9.01/8.90 (−1.24)

**Table 3:** City-averaged sensitivities of PNO_3_^−^ and PNH_4_^+^ concentration to NH_3_ emissions in July 2020 (unit: μg/m^3^).

	City	A Posteriori	A Priori		City	A Posteriori	A Priori
$S_{{NH}_{3}}^{{PNO}_{3}^{-}}$	Beijing	0.43	0.42	$S_{{NH}_{3}}^{{PNH}_{4}^{+}}$	Beijing	0.15	0.14
	Baoding, Langfang	0.64	0.60		Baoding, Langfang	0.20	0.18
	Cangzhou	0.86	0.72		Cangzhou	0.26	0.22
	Chengde	0.34	0.35		Chengde	0.21	0.19
	Handan	1.60	1.18		Handan	0.50	0.37
	Hengshui	0.82	0.81		Hengshui	0.24	0.24
	Qinhuangdao	0.79	0.82		Qinhuangdao	0.48	0.46
	Shijiazhuang	1.24	1.03		Shijiazhuang	0.38	0.32
	Tangshan	0.74	0.77		Tangshan	0.31	0.34
	Xingtai	0.46	0.50		Xingtai	0.14	0.15
	Zhangjiakou	0.36	0.30		Zhangjiakou	0.17	0.13
	Tianjin	0.65	0.58		Tianjin	0.20	0.19

**Table 4:** City-averaged sensitivities of PNO_3_^−^ and PNH_4_^+^ concentration to NO*x* emissions in July 2020 (unit: μg/m^3^).

	City	A Posteriori	A Priori		City	A Posteriori	A Priori
$S_{{NO}_{x}}^{{PNO}_{3}^{-}}$	Beijing	0.38	0.23	$S_{{NO}_{x}}^{{PNH}_{4}^{+}}$	Beijing	0.11	0.07
	Baoding, Langfang	0.82	0.38		Baoding, Langfang	0.25	0.12
	Cangzhou	0.60	0.30		Cangzhou	0.18	0.09
	Chengde	0.38	0.14		Chengde	0.10	0.04
	Handan	0.94	0.40		Handan	0.27	0.12
	Hengshui	0.46	0.30		Hengshui	0.14	0.09
	Qinhuangdao	0.60	0.45		Qinhuangdao	0.13	0.11
	Shijiazhuang	1.39	0.74		Shijiazhuang	0.41	0.22
	Tangshan	0.48	0.29		Tangshan	0.13	0.07
	Xingtai	0.40	0.21		Xingtai	0.12	0.06
	Zhangjiakou	0.49	0.12		Zhangjiakou	0.14	0.04
	Tianjin	0.49	0.31		Tianjin	0.14	0.09

**Table 5:** Concentration changes and relative reduction rates of PNO_3_^−^ and PNH_4_^+^ in the BTH region under different emission reduction scenarios. (Values outside the parentheses represent concentration changes, unit: μg/m^3^; values in parentheses represent relative reduction rates, unit: %).

Emission Reduction Scenarios	Prior		Posterior	
	PNO_3_^−^	PNH_4_^+^	PNO_3_^−^	PNH_4_^+^
70% NH_3_ reduction (S1)	−1.13 (−58)	−0.71 (−23)	−1.90 (−49)	−0.66 (−18)
30% NO*_x_* reduction (S2)	−0.42 (−21)	−0.26 (−8)	−0.87 (−22)	−0.25 (−7)
70% NH_3_ + 30% NO*_x_* co-reduction (S3)	−1.24 (−63)	−0.59 (−19)	−2.27 (−58)	−0.77 (−21)

## Data Availability

The original contributions to the study are included in the article; further inquiries can be directed to the corresponding author.

## Supplementary Materials

The following supporting information can be downloaded at: https://www.mdpi.com/article/10.3390/toxics14080657/s1, Figure S1: Comparison between simulated and satellite-observed NH_3_ and NO_2_ column concentrations in BTH Region in July 2020.

## Appendix A. Discrete Kalman Filter Multi-Species Joint Inversion Framework

### Basic Algorithm Principle

This study adopts a discrete Kalman filter (DKF) multi-species joint inversion method coupled with the CAMx-Direct Decoupled Method (CAMx-DDM). The DDM module quantifies linear spatial response relationships between precursor emissions and pollutant column concentrations to construct a sensitivity matrix. Combined with monthly satellite vertical column observations, iterative optimization is performed to resolve the non-linear coupling between NH_3_ and NO*_x_* emissions and ambient concentrations, quantify biases in the prior emission inventory, and generate corrected posterior emission estimates.

The iterative update formula for emission adjustment factors is written as Equation (A1):

(A1) $${{\bar{E}}_{i+1}={\bar{E}}_{i}+A}_{i}S_{matrix}^{\left(1\right)T}{\left(S_{matrix}^{\left(1\right)}A_{i}S_{matrix}^{\left(1\right)T}+R_{i}\right)}^{-1}({\bar{C}}_{matrix}^{obs}-{\bar{C}}_{matrix}^{mod}-S_{matrix}^{\left(1\right)}{\bar{E}}_{i})$$

$S_{matrix}$ is the multi-species sensitivity matrix (Equation (A2)), integrating self-sensitivity and cross-sensitivity between NH_3_ and NO*_x_* precursors:

(A2) $$S_{matrix}=\left[\begin{array}{c} \begin{array}{cc} S_{{NH}_{3}}^{{{NH}_{3}}^{\left(1,1\right)}} & S_{NOx}^{{{NH}_{3}}^{\left(1,1\right)}}\\ S_{{NH}_{3}}^{{{NH}_{3}}^{\left(1,2\right)}} & S_{NOx}^{{{NH}_{3}}^{\left(1,2\right)}} \end{array}\;\\ \begin{array}{cc} \vdots & \vdots \\ S_{{NH}_{3}}^{{NO2}^{\left(1,1\right)}} & S_{NOx}^{{NO2}^{\left(1,1\right)}} \end{array}\;\\ \begin{array}{cc} S_{{NH}_{3}}^{{NO2}^{\left(1,2\right)}} & S_{NOx}^{{NO2}^{\left(1,2\right)}}\\ \vdots & \vdots \end{array}\; \end{array}\right]$$

$C_{matrix}$ is the column vector stack of simulated and satellite-observed vertical densities (Equation (A3)):

(A3) $$C_{matrix}=\left[\begin{array}{c} \begin{array}{c} {{NH}_{3}}^{\left(1,1\right)}\\ {{NH}_{3}}^{\left(1,1\right)} \end{array}\\ \vdots \\ {{NO}_{2}}^{\left(1,1\right)}\\ {{NO}_{2}}^{\left(1,1\right)}\\ \vdots \end{array}\right]$$

In the above equations (Equations (A1)–(A3)), ${\bar{E}}_{i+1}$ denotes the updated emission adjustment factor, and ${\bar{E}}_{i}$ denotes the prior emission adjustment factor. $S_{matrix}$ represents the sensitivity matrix. Among its terms, $S_{{NH}_{3}}^{{NH}_{3}}$ and $S_{{NO}_{x}}^{{NO}_{2}}$ refer to the sensitivity of NH_3_ concentrations to NH_3_ emissions and the sensitivity of NO_2_ concentrations to NO*_x_* emissions, respectively. Cross-interactions between NH_3_ and NOx are also incorporated: $S_{{NO}_{x}}^{{NH}_{3}}$ is the sensitivity of NH_3_ concentrations to NO*_x_* emissions, while $S_{{NH}_{3}}^{{NO}_{2}}$ represents the sensitivity of NO_2_ concentrations to NH_3_ emissions.

${\bar{C}}_{matrix}^{obs}$ and ${\bar{C}}_{matrix}^{mod}$ stand for the matrix of satellite-observed and model-simulated vertical column densities of NH_3_ and NO_2_, with units of molec/cm^2^. The satellite data in ${\bar{C}}_{matrix}^{obs}$ are monthly vertical column products; accordingly, we vertically integrate the simulated concentration and sensitivity outputs and compute monthly mean values as inputs for inversion calculations.

The prior emission covariance matrix $A_{i}$ follows $A_{i}={\left(\sigma_{i}^{E}\right)}^{2}$, where $\sigma_{i}^{E}$ is the relative uncertainty of prior precursor emissions. The prior uncertainty for NO*_x_* emissions is set to 10% ($\sigma_{i}^{E_{NO_{x}}}$), as reported in Refs. [25,26], and the prior uncertainty for NH_3_ emissions is set to 50% ($\sigma_{i}^{E_{{NH}_{3}}}$) according to Ref. [10].

Observation errors within satellite datasets include instrumental noise and other unquantifiable uncertainties. To prevent numerical instability during inversion, the observation error covariance matrix is defined as $R_{i}=max{\left[\sigma_{min},\;\;C_{matrix}^{obs}\times \chi^{obs}\right]}^{2}$. Here, $\sigma_{min}$ is the minimum error threshold (unit: molec/cm^2^). Following official satellite documentation and existing studies [10,25], $\sigma_{min}$ is assigned as 0.30 × 10^15^ molec/cm^2^ for TROpospheric Monitoring Instrument NO_2_ vertical columns and 1.00 × 10^16^ molec/cm^2^ for Infrared Atmospheric Sounding Interferometer NH_3_ vertical columns. $\chi^{obs}$ is a scaling factor for satellite retrieval uncertainty, varying from 0 to 2 throughout the inversion to account for seasonal variations in NH_3_ and NO*_x_* emissions, which guarantees numerical stability and physically reasonable inversion results. Both $A_{i}$ and $R_{i}$ are assumed to be diagonal matrices in this work.
