# Supplementary material for: Responses of Secondary Inorganic Aerosols to Synergistic NOx and NH3 Emission Control Based on an Inversion Inventory
Source: Toxics. 2026 Jul 26;14(8):657. doi: 10.3390/toxics14080657 (PMC13517181; doi:10.3390/toxics14080657)
Supplement: Supplementary file 1 [file toxics-14-00657-s001.zip › toxics-4373695-supplementary.pdf]

Satellite retrievals and model simulations before and after joint inversion were extracted for column concentrations of gaseous NH_3 and NO_2 over the Beijing–Tianjin–Hebei (BTH) region to evaluate model performance under prior and posterior emission inventories.

From the overall statistical indicators of the region in July 2020 (Figure S1a), the simulation effect of NO_2 column concentration after joint inversion has also improved. After inversion based on satellite data, the R value increased from 0.75 before inversion to 0.77, and the RMSE decreased from 2.4×10^{15} molec/cm^2 to 1.7×10^{15} molec/cm^2. The monthly average NH_3 column concentration value and the R value based on IASI observations increased from 0.70 before inversion to 0.72, indicating that the consistency between the simulated concentration and the satellite observation improved. However, the RMSE increased from 3.6×10^{15} molec/cm^2 to 1.3×10^{16} molec/cm^2. This is because this study adopted a unified emission correction coefficient for all cities in the BTH region. While correcting the underestimated bias of the prior emissions and improving spatial correlation overall, it also amplified the simulation errors of some local extreme grids. (Figure S1b).

Overall, joint inversion of emissions narrows the overall discrepancies between simulated and satellite-observed NH_3 and NO_2 column concentrations, demonstrating that the inversion-optimized emission inventory effectively enhances model performance in reproducing column abundance. Meanwhile, our results reveal limitations of the uniform correction framework in resolving fine-scale, intense local emission hotspots, which provides a basis for future refined grid-by-grid inversion strategies.

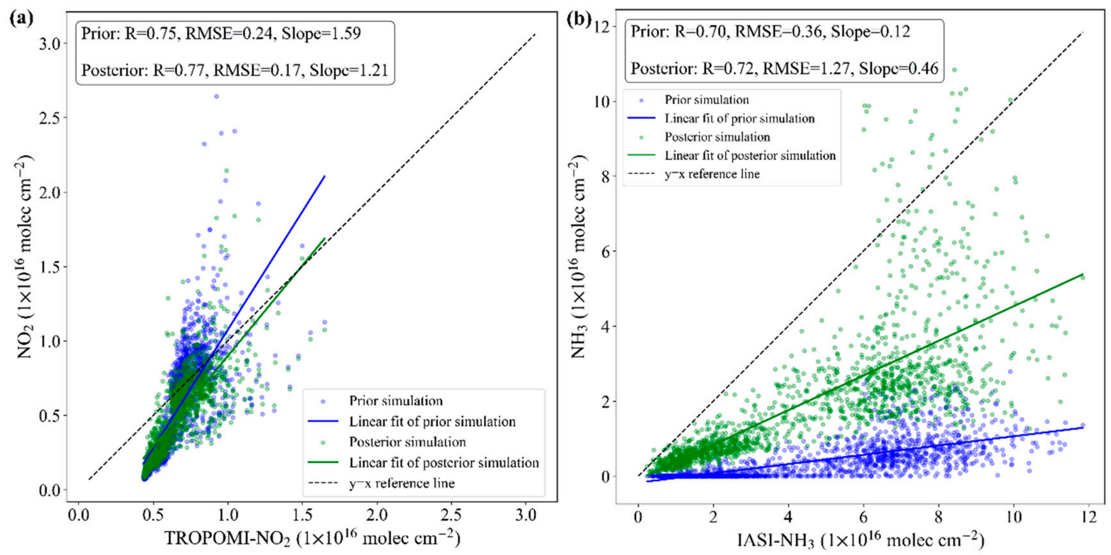

Figure S1. Comparison between simulated and satellite-observed NH_3 and NO_2 column concentrations in BTH Region in July 2020.
